# Radiomics Analysis of Contrast-Enhanced CT for the Preoperative Prediction of Microvascular Invasion in Mass-Forming Intrahepatic Cholangiocarcinoma

**DOI:** 10.3389/fonc.2021.774117

**Published:** 2021-11-19

**Authors:** Fei Xiang, Shumei Wei, Xingyu Liu, Xiaoyuan Liang, Lili Yang, Sheng Yan

**Affiliations:** ^1^ Department of Hepatobiliary and Pancreatic Surgery, The Second Affiliated Hospital, Zhejiang University School of Medicine, Hangzhou, China; ^2^ Department of Pathology, The Second Affiliated Hospital, Zhejiang University School of Medicine, Hangzhou, China; ^3^ Department of Radiology, The First Affiliated Hospital, Zhejiang University School of Medicine, Hangzhou, China

**Keywords:** intrahepatic cholangiocarcinoma, microvascular invasion, radiomics, computed tomography, nomogram

## Abstract

**Background:**

Microvascular invasion (MVI) has been shown to be closely associated with postoperative recurrence and metastasis in patients with intrahepatic cholangiocarcinoma (ICC). We aimed to develop a radiomics prediction model based on contrast-enhanced CT (CECT) to distinguish MVI in patients with mass-forming ICC.

**Methods:**

157 patients were included and randomly divided into training (n=110) and test (n=47) datasets. Radiomic signatures were built based on the recursive feature elimination support vector machine (Rfe-SVM) algorithm. Significant clinical-radiologic factors were screened, and a clinical model was built by multivariate logistic regression. A nomogram was developed by integrating radiomics signature and the significant clinical risk factors.

**Results:**

The portal phase image radiomics signature with 6 features was constructed and provided an area under the receiver operating characteristic curve (AUC) of 0.804 in the training and 0.769 in the test datasets. Three significant predictors, including satellite nodules (odds ratio [OR]=13.73), arterial hypo-enhancement (OR=4.31), and tumor contour (OR=4.99), were identified by multivariate analysis. The clinical model using these predictors exhibited an AUC of 0.822 in the training and 0.756 in the test datasets. The nomogram combining significant clinical factors and radiomics signature achieved satisfactory prediction efficacy, showing an AUC of 0.886 in the training and 0.80 in the test datasets.

**Conclusions:**

Both CECT radiomics analysis and radiologic factors have the potential for MVI prediction in mass-forming ICC patients. The nomogram can further improve the prediction efficacy.

## Introduction

Intrahepatic cholangiocarcinoma (ICC) arises from the epithelial cells of the intrahepatic bile ducts and is the second leading primary liver malignancy (1). ICC can be classified into intraductal, periductal infiltrative, and mass-forming types with macroscopic growth classification. The mass-forming ICC is the most predominant type, accounting for 80% to 90% of all ICC cases (2, 3). According to statistics, the incidence and mortality of ICC are continuously increasing worldwide over the years (4, 5). Surgery is the mainstay of therapy for localized, resectable ICC; Nevertheless, the prognosis remains unsatisfactory, with 5-year overall survival ranging from 15% to 23% (6, 7). High rates of recurrence and metastasis following resection are the leading causes of poor prognosis. In fact, recurrence is estimated to occur in 45% to 70% of patients with ICC (7–10).

Studies have revealed that microvascular invasion (MVI) is a considerable poor-prognostic factor in ICC. MVI has been shown to be closely associated with postoperative recurrence and metastasis (11–13). Tsukamoto et al. (14) reported that the absence of MVI and lymph node metastasis were the only two independent factors for recurrence-free survival over 5 years after liver resection for ICC. Hu et al. (15) showed that ICC patients with MVI exhibited enhanced aggressive behavior with a higher incidence of adjacent tissue infiltration, organ invasion, and satellite lesions; In addition, patients with MVI had a significantly worse disease-free survival (DFS) than patients without MVI. Moreover, Ercolani et al. (16) demonstrated that patients without MVI significantly experienced favorable median overall survival (OS) time than those patients with MVI in all types of cholangiocarcinoma. In contrast to macrovascular invasion, which can be evaluated by radiologic images, MVI can only be detected by postoperative pathological examination, limiting its value in the clinical setting. Recently, some studies have been performed to identify preoperative predictive markers for ICC patients with MVI. Laboratory parameters, including routine blood tests, liver function, and cancer biomarkers such as ALT, AFP, CA-199, have been screened and defined as predictive indicators (17, 18). However, despite the relative ease of obtaining these data, some parameters are controversial and have to be systematically evaluated in clinical practice. Radiological characteristics, such as tumor morphology, arterial phase enhancement pattern, tumor diameter, and apparent diffusion coefficient (ADC) values, were associated with MVI in ICC patients (18, 19). However, imaging features were assessed subjectively and may lead to interobserver variability.

Radiomics, an omics-based approach allowing for the extraction of quantitative features from raw medical images, has been used to perform objective and quantitative analysis of tumor heterogeneity and cancer phenotype (20). Radiomics has been widely utilized in predicting MVI for patients with hepatocellular carcinoma (HCC), and its feasibility and potential benefits have been proved (21–23). However, few studies have evaluated the prediction value of radiomics for ICC. Zhou et al. (24) exacted features from MRI images and a fusion radiomics signature comprising seven features was established for MVI prediction in ICC patients with an area under the receiver operating characteristic curve (AUC) of 0.85. Contrast-enhanced CT (CECT) is the most common imaging modality for diagnosis and assessment of ICC; however, there are currently no studies that evaluated the radiomics analysis of CECT for MVI prediction in patients with ICC.

Therefore, we aimed to verify whether radiomics analysis based on CECT could be useful to predict MVI in mass-forming ICC. Additionally, clinical-radiologic predictors were also evaluated and compared with radiomics analysis.

## Materials and Methods

### Patient Characteristics

**Figure 1:**
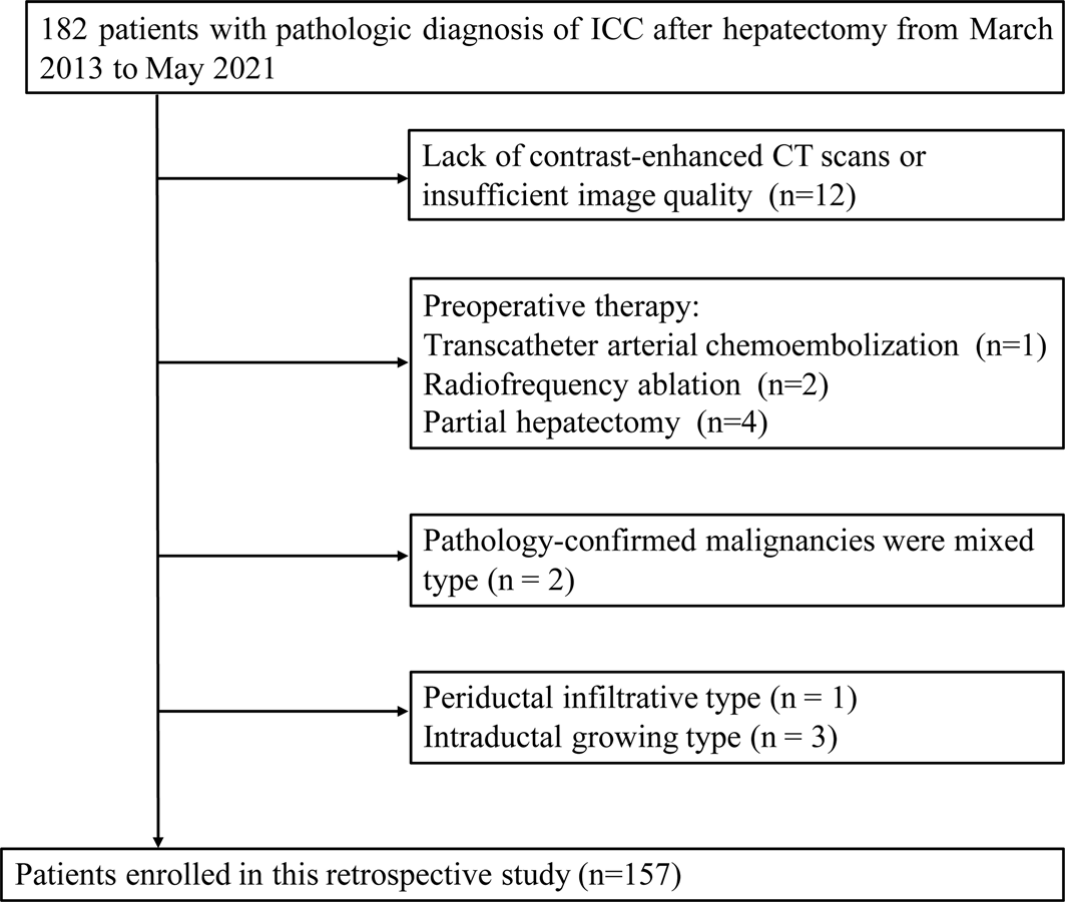
Flow chart of patients recruitment in this study.

182 patients with pathologic diagnosis of ICC after hepatectomy were retrospectively identified in our hospital from March 2013 to May 2021. Inclusion criteria were as follows: (1) Malignancies were classified as mass-forming ICC; (2) CECT scans performed within two weeks before surgery; (3) MVI status was described on pathology reports. Exclusion criteria were: (1) Pathology-confirmed malignancies were mixed, periductal infiltrative or intraductal growing types of ICC; (2) prior intervention or partial hepatectomy; (3) Lack of contrast-enhanced CT scans or insufficient image quality; (4) Grossly tumor thrombus in the portal vein or bile duct tumor thrombosis. The detailed selection process is described in **Figure 1**. Of the 182 screened patients,157 patients were finally enrolled and randomly assigned to a training dataset (n=110) and a test dataset (n=47), with a split ratio of 7:3. The Ethics committee of our hospital approved the present retrospective study.

### Clinical Characteristics and Radiological Evaluation

Demographic covariates were collected and compared between the MVI group and non-MVI group both in training and test datasets, including age, sex, hepatitis B immunology, platelet count (PLT), serum albumin (ALB), serum direct bilirubin (DB), serum total bilirubin (TB), γ-glutamyl transpeptidase (GGT), alkaline phosphatase (ALP), serum alanine aminotransferase (ALT), aspartate aminotransferase (AST), international normalized ratio (INR), prothrombin time (PT), carcinoembryonic antigen (CEA), Carbohydrate antigen199 (CA-199). Radiological features, including the number of segments involved, satellite nodules, lymph node status, intrahepatic duct dilatation, tumor contour, arterial rim enhancement, arterial hypo-enhancement, intratumor vascularity, hepatic capsular retraction, were blindly evaluated and recorded by two readers. The detail of radiological evaluation and our protocol for CECT scan acquisition is described in the **Supplementary Presentation**.

### Histology

All resected specimens were examined and cross-checked by at least two senior pathologists. Seven tissues were harvested and examined for MVI diagnosis from the resected specimen, including the central and four sides of tumor tissues and two adjacent non-carcinoma tissues. MVI was defined as the invasion of tumor emboli into a vascular space that only can be detected on microscopy (25).

### Radiomic Feature Extraction

**Figure 2:**
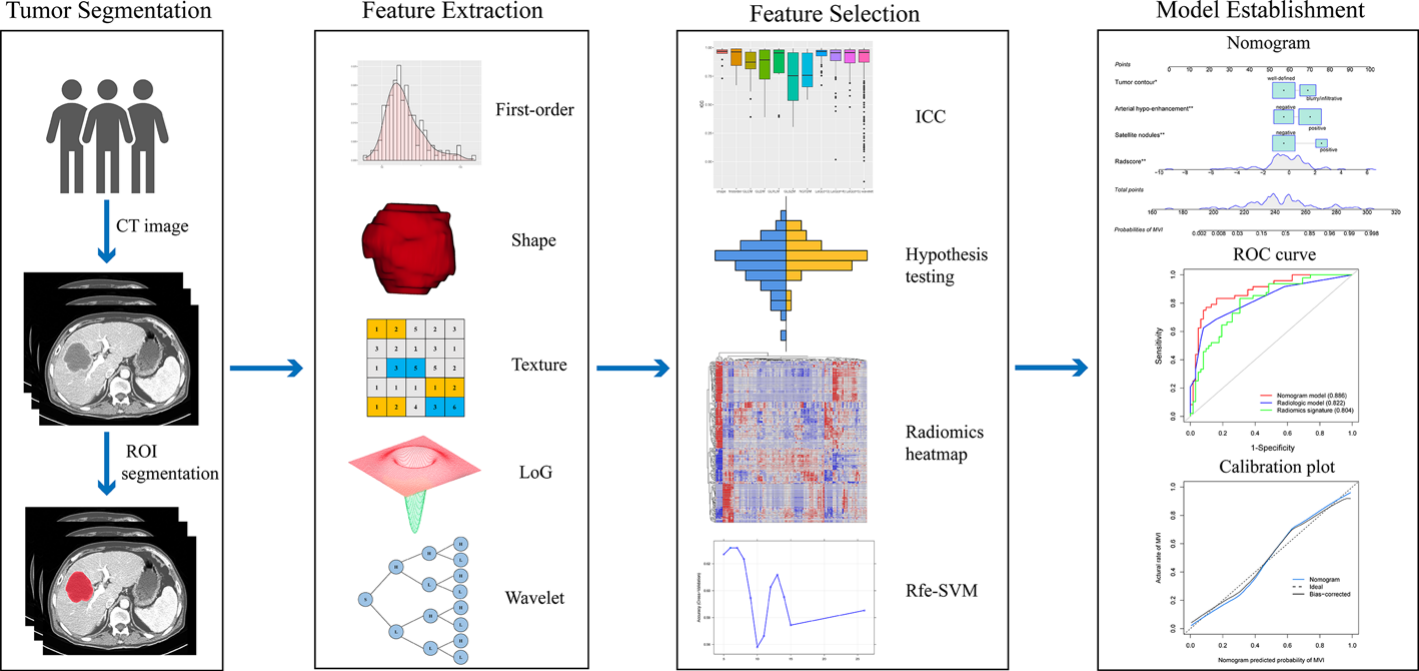
Workflow for the radiomics process. After CT images were acquired, segmentation of the tumor was performed. The extracted radiomics features include first-order, shape, Laplacian of Gaussian, texture, and wavelet features. A four-step approach was performed for feature dimension reduction. Intra- and inter-class correlation coefficient (ICC) was used to evaluate the reproductivity of features. Values lower than 0.8 were eliminated. Student’s t-test or Mann–Whitney-U test was performed to find the differential radiomics features. A heatmap shows the Pearson correlation coefficients matrix among radiomics features. Rfe-SVM was applied to develop the radiomics signature. A nomogram was constructed by integrating independent radiological predictors and portal phase image radiomics signature. The nomogram and radiologic model’s discriminative ability were compared with the ROC curve analysis and quantified by the AUC. The calibration curve demonstrated good agreement between the actual and nomogram predicted probabilities.

The radiomics workflow is depicted in **Figure 2**. Region of interests (ROIs) of the whole tumor was contoured on arterial and portal venous phase of CT images using ITK-SNAP software (The specific ROIs segmentation was shown in the **Supplementary Presentation**). The Pyradiomics toolkit was used to extract features from each three-dimensional ROI (26). For each phase, 1130 radiomics features were extracted, including 18 first-order features, 14 shape features, 75 textural features, 279 Laplacian of Gaussian features (sigma=3.0,4.0,5.0), and 744 wavelet features. In total, 2260 features derived from arterial and portal phases were obtained for each patient. The parameter setting for radiomics features extraction and the detailed radiomics features is depicted in the **Supplementary Presentation** and **Supplementary Table**.

### Radiomics Signature Construction

Before feature dimension reduction, values of radiomics features were transformed to a normal distribution by the z-score method. Then, feature dimension reduction and selection were performed in a four-step approach. Firstly, the intra- and inter-class correlation coefficient was used to evaluate the repeatability of radiomics features between the intra- and inter-observer. Features with intraclass and interclass correlation coefficients lower than 0.80 were eliminated. The specific process was described in the **Supplementary Presentation**. Secondly, Mann–Whitney-U test or Student’s t-test was applied to find the differential radiomics features with effects of p < 0.05 were considered statistically significant. Thirdly, Pearson correlation coefficient > 0.75 was used as a cutoff to eliminate the highly correlated features. Lastly, a recursive feature elimination support vector machine (Rfe-SVM) method was performed to construct the radiomics signature. The formula of the radiomics signature was displayed in the **Supplementary Presentation**.

According to the process set up above, three radiomics signatures were developed based on features exacted from the arterial phase, portal phase, and pooling features of the two phases. Receiver operating characteristic (ROC) analysis was performed to assess the three signatures’ predictive capability.

### Development of the Clinical Model and the Nomogram

For constructing the clinical model, univariate analysis was applied to identify independent risk factors (including clinical characteristics and CECT radiological features) between the MVI and the non-MVI groups. The multivariate logistic model was built with significant factors (p<0.05) from the univariate analysis as inputs. Odds ratios (OR) and their 95% confidence intervals (CI) were used to express the estimate relative risk. The clinical-radiomics nomogram was built using the optimal radiomics signature and independent clinical-radiologic risk factors in the clinical model. Variance inflation factor (VIF) coefficients were examined to check the collinearity. Comparison of the ROC curves among nomogram model, radiomics signature, and clinical model was performed by using DeLong’s test. A calibration curve was plotted to evaluate the calibration of the nomogram.

### Statistical Analysis

The Chi-square test or Fisher exact test was used for categorical variables comparison. For continuous variables, Student’s t-test or Mann-Whitney U test was used. We considered p < 0.05 (two-tailed) as statistically significant. Statistical analysis was conducted with SPSS (v. 26.0), R software (v. 3.6.1), and Python (v. 3.9.2).

## Results

### Patient Characteristics

The baseline clinical-radiological characteristics are demonstrated in **Table 1**. The incidence of MVI between the training and test datasets shows no statistical difference (48/110 *vs*. 20/47, p=0.900). Patients with MVI were associated with larger tumor size, more liver segments invasion, and blurry or infiltrative tumor contour, which was confirmed in the training and test datasets. Significant differences were found in terms of age, PLT, satellite nodules, lymph node metastasis and arterial hypo-enhancement in the training dataset but not validated in the test dataset. Other baseline variables did not differ between the training and test datasets.

**Table 1:** Baseline characteristics of patients.

Characteristics	Training dataset			Test dataset		
	MVI-positive (n=48)	MVI-negative (n=62)	p value	MVI-positive (n=20)	MVI-negative (n=27)	p value
Age, years, mean ± SD	61.2 ± 8.8	65.6 ± 8.5	0.008	56.0 ± 12.6	57.5 ± 8.8	0.616
Sex			0.413			0.706
male	27 (56.3)	30 (48.4)		10 (50)	15 (55.6)	
female	21(43.7)	32 (41.6)		10 (50)	12(44.4)	
HBV infection			0.682			0.209
Present	10 (20.8)	11 (17.7)		6 (30.0)	4 (14.8)	
absent	38 (79.2)	51(82.3)		14 (70.0)	23 (85.2)	
PLT, 10^9^/L, mean ± SD	202.3 ± 56.6	173.5 ± 70.2	0.023	243.8 ± 116	203.1 ± 68.8	0.173
Alb, g/L, median (IQR)	40.7 (37.1-43.2)	40.0 (36.6-42.7)	0.341	40.4 (35.6-43.6)	42.1 (37.8-44.0)	0.890
TBIL, μmol/L, median (IQR)	13.9 (10.4-23.3)	13.8 (10.6-18.4)	0.109	11.7 (8.7-17.8)	11.1 (9.2-13.2)	0.309
DBIL, μmol/L, median (IQR)	3.0 (2.2-4.9)	2.9 (2.3-4.0)	0.091	2.4 (2.0-5.8)	2.3 (2-2.9)	0.258
ALT, U/L, median (IQR)	27.5 (19.3-48)	23 (15.8-31.3)	0.159	27 (16.5-52.5)	21 (14-36)	0.477
AST, U/L, median (IQR)	31.5 (21.3-48.8)	28 (22-35.6)	0.223	27 (22.3-47.3)	27 (20-34)	0.406
ALP, U/L, median (IQR)	118.5 (90-225.5)	95.5 (76.8-140.8)	0.202	141.5 (106.3-315.0)	134 (111-145)	0.152
GGT, U/L, median (IQR)	81.5 (42.3-179)	46 (31.8-76.3)	0.099	71.5 (55.8-200.8)	83 (44-208)	0.801
PT, mean ± SD	13.0 ± 1.0	13.3 ± 1.0	0.119	13.0 ± 1.5	13.1 ± 1.1	0.959
INR, mean ± SD	1.0 ± 0.10	1.0 ± 0.09	0.603	1.0 ± 0.3	1.0 ± 0.1	0.481
CEA> 5 ug/L	15 (31.3)	17 (27.4)	0.661	9 (45.0)	7 (25.9)	0.172
CA-199>37 ug/L	32 (66.7)	32 (51.6)	0.112	17 (85.0)	12 (44.5)	0.005
Tumor size	5.9 ± 2.6	4.4 ± 2.1	0.002	6.4 ± 2.1	4.6 ± 2.0	0.004
Liver cirrhosis	10 (20.8)	9 (14.5)	0.385	2 (10.0)	5 (18.5)	0.417
No. of segments involved			0.005			0.009
Single	26 (54.2)	50 (80.6)		9 (45.0)	22 (81.5)	
Two or more	22 (45.8)	12 (19.4)		11 (55.0)	5 (18.5)	
Satellite nodules	20 (41.7)	3 (4.8)	<0.001	7 (35.0)	5 (18.5)	0.200
lymph node metastasis	27 (56.3)	8 (12.9)	<0.001	13 (65.0)	11 (40.7)	0.100
Intrahepatic duct dilatation	14 (29.2)	16 (25.8)	0.695	8 (40.0)	7 (25.9)	0.306
Tumor contour			<0.001			0.002
Well-defined	21 (43.8)	52 (83.9)		5 (25.0)	19 (70.4)	
Blurry/infiltrative	27 (56.2)	10 (16.1)		15 (75.0)	8 (29.6)	
Arterial rim- enhancement	13 (27.1)	27 (43.5)	0.075	6 (30.0)	9 (33.3)	0.808
Arterial hypo-enhancement	34 (70.8)	28 (45.2)	0.007	14 (70.0)	17 (63.0)	0.615
Intratumor vascularity	27 (56.3)	34 (54.8)	0.883	5 (25.0)	5 (18.5)	0.591
Hepatic capsular retraction	11 (22.9)	14 (22.6)	0.967	2 (10.0)	6 (22.2)	0.270

MVI, microvascular invasion; HBV, hepatitis B virus; PLT, platelets; Alb, albumin; TBIL, total bilirubin; DBIL, direct bilirubin; ALT, alanine aminotransferase; AST, aspartate transaminase; ALP, alkaline phosphatase; GGT, γ-glutamyl transpeptidase; PT, prothrombin time; INR, international normalized ratio; CEA, carcinoembryonic antigen; CA-199, cancer antigen 19-9; SD, standard deviation; IQR, interquartile range.

### Radiomics Signature Construction

A total of 1130 radiomics features were identified from each imaging phase. For features identified from the arterial phase image, 319 features were excluded with intraclass and interclass correlation coefficients lower than 0.8 (**Figure S1**). Then, 811 features were subjected to statistical hypothesis testing with Student’s t-test or Mann–Whitney-U test; 276 features were found significantly different between the MVI positive and non-MVI groups. Pearson correlation analysis found 254 features were highly correlated (correlation coefficient > 0.75) and were eliminated. Only 22 features were kept and subjected to Ref-SVM. Finally, 5 features were selected, and the arterial phase image radiomics signature was built.

**Figure 3:**
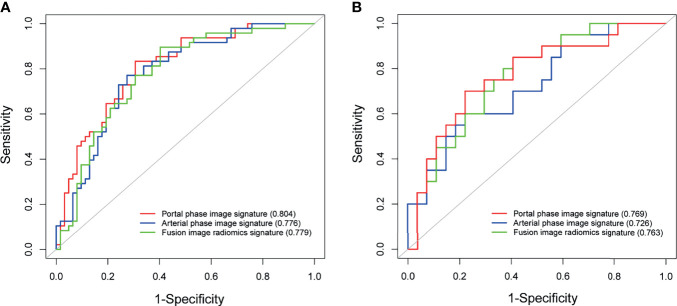
Predictive performance of radiomic signatures for microvascular invasion. ROC curves of radiomic signatures in the training dataset **(A)**. ROC curves of radiomic signatures in the test dataset **(B)**.

The portal phase image radiomics signature with 6 features and fusion radiomics signature with 12 features were built with a similar process. The specific flow and the selected features of three radiomic signatures were shown in the **Figure S2** and **Table 2**. The arterial phase image radiomics signature, portal phase image radiomics signature, and fusion radiomics signature showed good discriminative abilities for MVI prediction, with AUCs of 0.776 (95% CI 0.688–0.863), 0.804 (95% CI 0.723–0.885), and 0.779 (95% CI 0.692–0.865) in the training dataset, and AUCs of 0.726 (95% CI 0.581–0.871), 0.769 (95% CI 0.630–0.908), and 0.763 (95% CI 0.627–0.898), in the test dataset (**Figure 3**). The portal phase image radiomics signature achieved slightly better predictive performance than the other two radiomics signatures, but no statistical differences were found.

**Table 2:** The list of selected features in three radiomics signatures.

Signature	Features selected	Feature name
Arterial phase image signature	5	log-sigma-5-0-mm-3D_firstorder_Variance
		wavelet-LHL_glcm_InverseVariance
		wavelet-LHL_gldm_DependenceVariance
		wavelet-HLH_glcm_Correlation
		wavelet-HHL_glszm_SizeZoneNonUniformity
Portal phase image signature	6	original_firstorder_Skewness
		wavelet-LLH_glcm_Correlation
		wavelet-HLL_glcm_InverseVariance
		wavelet-HHL_glszm_SizeZoneNonUniformity
		wavelet-LLL_glcm_Imc1
		wavelet-LLL_ngtdm_Strength
Fusion radiomics signature	12	PP_original_firstorder_Skewness
		PP_log-sigma-3-0-mm-3D_glszm_GrayLevelVariance
		PP_wavelet-LLH_glcm_Correlation
		PP_wavelet-HLL_glszm_LargeAreaLowGrayLevelEmphasis
		PP_wavelet-HLH_glcm_InverseVariance
		PP_wavelet-HHL_glszm_SizeZoneNonUniformity
		PP_wavelet-LLL_ngtdm_Strength
		AP_log-sigma-4-0-mm-3D_glszm_LargeAreaHighGrayLevelEmphasis
		AP_log-sigma-5-0-mm-3D_firstorder_Kurtosis
		AP_wavelet-LHL_glrlm_GrayLevelNonUniformityNormalized
		AP_wavelet-LLL_firstorder_Mean
		AP_wavelet-LLL_glcm_Contrast

PP, portal phase; AP, arterial phase.

Compared to non-MVI group, the MVI positive group had a significantly higher portal phase image Rad-score [median (IQR)] in the training dataset [0.549 (-0.302∼1.244) *vs* −0.916 (-1.829∼-0.050), P < 0.001]. Consistent results were obtained in the test dataset [1.151 (-0.075∼1.855) *vs* −0.505 (-1.032∼0.511), P=0.003] (**Figure S3**).

### Development of the Radiologic Model and Nomogram

Among all baseline variables, three significant predictors including satellite nodules [OR=13.73 (3.14–59.93), P<0.001], arterial hypo-enhancement [OR=4.31 (1.55–11.94), P=0.005], and tumor contour [OR=4.99 (1.76–14.18), P=0.003] were identified in the training dataset by univariate analysis and further confirmed in multivariate analysis (**Table 3**). The radiologic model developed with the three independent risk factors exhibited AUC of 0.822 (95% CI: 0.741–0.903) and 0.756 (95% CI: 0.616–0.895) in the training and test datasets.

**Table 3:** Univariate and multivariate analyses of risk factors for MVI.

Variables	Univariate analysis			Multivariate analysis		
	OR	95%CI	P-value	OR	95% CI	P-value
Age (≥50 vs <50)	1.067	0.096-11.837	0.958			
Sex (male vs female)	0.571	0.118-2.762	0.486			
HBV infection	6.761	0.841-54.374	0.072			
Alb (>40 vs ≤ 40)	1.712	0.387-7.572	0.479			
TBIL (>50 vs ≤ 50)	2.087	0.329-13.235	0.435			
DBIL (>6.8 vs ≤ 6.8)	5.270	0.251-110.61	0.285			
ALT (>50 vs ≤ 50)	13.122	0.658-261.57	0.092			
AST (>40 vs ≤ 40)	0.219	0.017-2.756	0.240			
ALP (>125 vs ≤ 125)	1.619	0.174-15.069	0.672			
GGT (>50 vs ≤ 50)	1.214	0.206-7.149	0.830			
PT (>13 vs ≤ 13)	0.902	0.077-10.608	0.195			
INR (per 0.1 increase)	0.208	0.012-3.619	0.281			
(>1.0 vs ≤ 1.0)						
PLT	1.006	0.995-1.017	0.256			
CEA (>5 vs ≤ 5)	0.779	0.129-4.688	0.785			
CA-199 (>37 vs ≤ 37)	1.469	0.371-5.820	0.584			
Tumor size	0.990	0.953-1.029	0.617			
Cirrhosis	0.967	0.086-10.835	0.978			
No. of segments involved (single vs two/more)	2.244	0.508-9.519	0.287			
Satellite nodules	33.154	2.689-408.79	0.006	13.726	3.144-59.93	<0.001
lymph node metastasis	4.386	0.906-21.247	0.066			
Intrahepatic duct dilatation	0.252	0.034-1.882	0.179			
Tumor contour (well-defined vs blurry/infiltrative)	7.535	1.267-42.660	0.026	4.992	1.757-14.18	0.003
Arterial rim- enhancement	2.135	0.315-14.443	0.437			
Arterial hypo-enhancement	20.298	2.365-174.22	0.006	4.308	1.554-11.94	0.005
Intratumor vascularity	0.814	0.213-3.120	0.764			
Hepatic capsular retraction	1.400	0.256-7.662	0.698			

MVI, microvascular invasion; HBV, hepatitis B virus; Alb, albumin; TBIL, total bilirubin; DBIL, direct bilirubin; ALT, alanine aminotransferase; AST, aspartate transaminase; ALP, alkaline phosphatase; OR, odds ratios; GGT, γ-glutamyl transpeptidase; CI, confidence intervals; PT, prothrombin time; INR, international normalized ratio; PLT, platelets; CEA, carcinoembryonic antigen; CA-199, cancer antigen 19-9.

**Figure 4:**
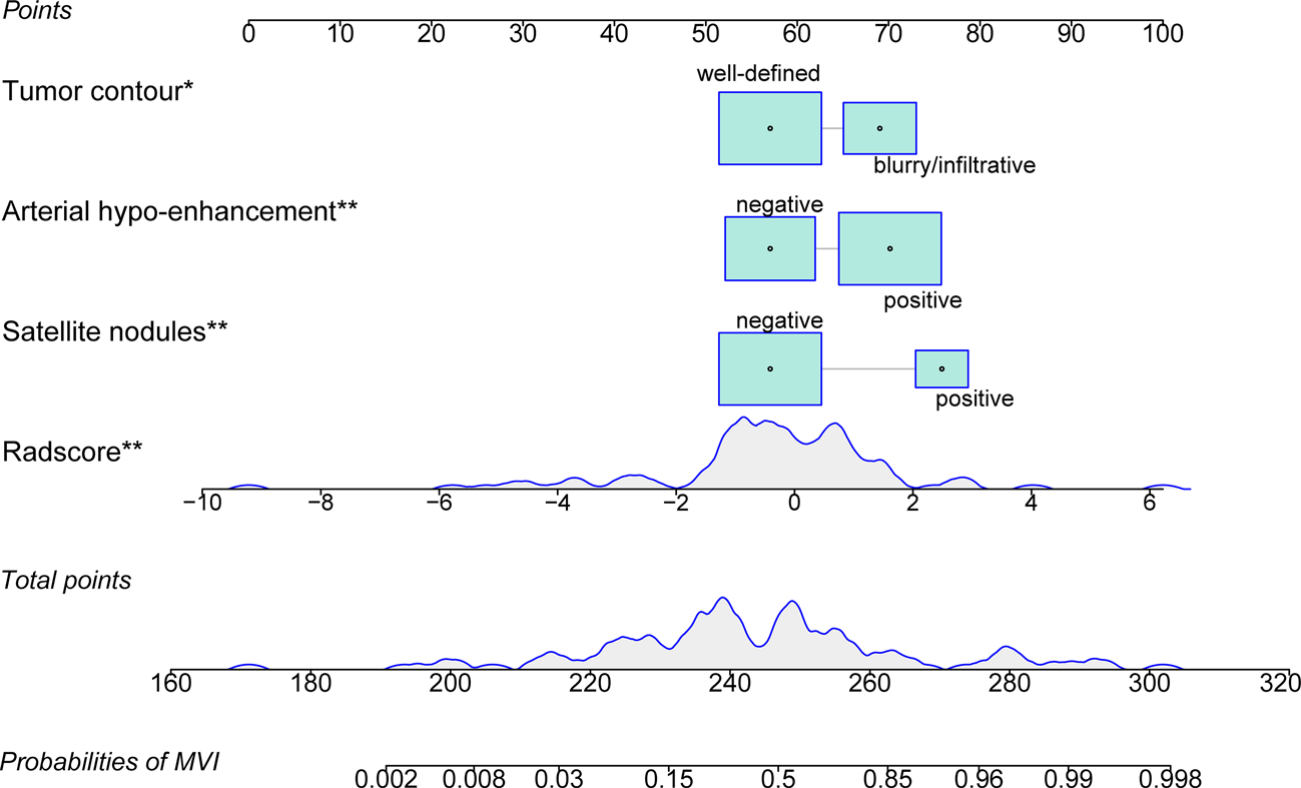
The radiomics nomogram was developed by incorporating the portal phase image radiomics signature, satellite nodules, arterial hypo-enhancement, and tumor contour. *p < 0.05; **p < 0.01.

**Figure 5:**
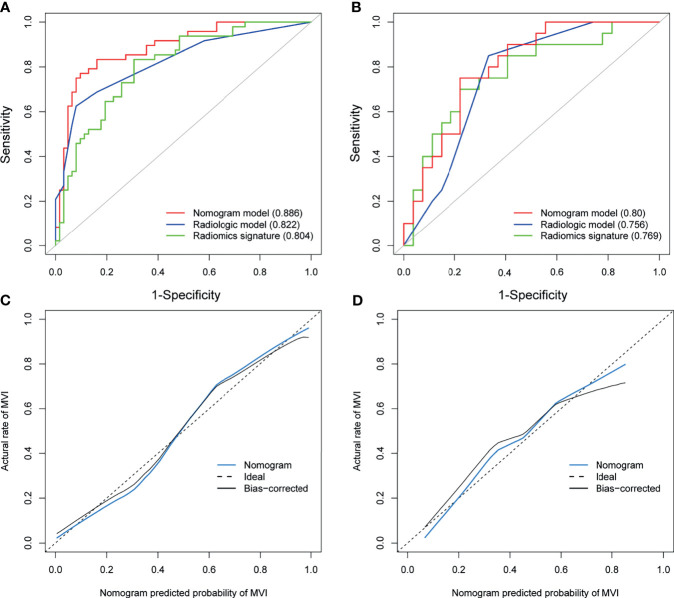
Assessing the discriminative performance of the nomogram and comparison with other predictive models. The nomogram showed a significantly higher discriminative power than the radiomics signature and the radiologic model for the prediction of microvascular invasion in the training dataset **(A)**, but did not differ in the test dataset **(B)**. The calibration plots demonstrate that the nomogram-predicted probabilities were consistent with actual MVI incidence in the training **(C)** and test **(D)** datasets.

We generated the nomogram using the independent predictors in the radiologic model and portal phase image radiomics signature with logistic regression (**Figure 4**). The VIFs for satellite nodules, arterial hypo-enhancement, tumor contour, and portal phase image radiomics signature were less than 10 (satellite nodules: 1.11; arterial hypo-enhancement: 1.14; tumor contour: 1.02; radiomics signature: 1.03), suggesting no collinearity between these variables. The nomogram demonstrated satisfactory prediction efficacy, with an AUC of 0.886 (95% CI: 0.823–0.949) and 0.80 (95% CI: 0.675–0.925) in the training and test datasets. The specific performances of nomogram are shown in **Table 4**. In the training dataset, the nomogram achieved higher AUC than the radiologic model (P =0.011) and portal phase image signature (P = 0.019) (**Figure 5A**). However, there were no statistical differences in the test dataset (nomogram vs radiologic model, portal phase image radiomics signature; P = 0.322, P = 0.642, respectively) (**Figure 5B**).

**Table 4:** Performance of nomogram for MVI prediction.

Group	Sensitivity (%)	Specificity (%)	Accuracy (%)	PPV (%)	NPV (%)	AUC (95%CI)	Cut-off
Training dataset	77.1 (37/48)	90.3 (56/62)	84.5 (93/110)	86.0 (37/43)	83.5 (56/67)	0.886 (0.823–0.949)	>0.157
Test dataset	77.8 (21/27)	75.0 (15/20)	76.6 (36/47)	71.4 (15/21)	80.8 (21/26)	0.800 (0.675–0.925)	>0.157

PPV, positive predictive value; NPV, negative predictive value; AUC, area under the curve.

The calibration plots (**Figures 5C, D**) were consistent between the nomogram prediction and the actual observed probability. Decision curve analysis (**Figure S4**) revealed that the nomogram achieved highest net benefit compared with the radiologic model and radiomics signature. The Hosmer-Lemeshow test showed no significant both in the training (p=0.206) and test datasets (p=0.529), indicating the nomogram was well fitted. A representative case showing the discriminative ability of the nomogram is depicted in **Figure S5**.

## Discussion

With increasing recognition of MVI and its prognostic value after hepatectomy, preoperative MVI prediction has become a research hotspot in recent years. In fact, MVI status has been considered as an important event for preoperative surgical decision-making in China. Some studies recommend that anatomical liver resection or wide resection margin is a priority for HCC patients with MVI (27–30). In ICC patients, many studies suggested that a wide surgical margin may lead to favorable survival benefits (7, 31–33). Hu et al. (32) reported that very early recurrence (defined as recurrence within 6 months) after ICC hepatectomy mostly occurred in the surgical margin site. We think that MVI plays an important role in early recurrence after ICC hepatectomy, because MVI generally occurs on the tumor edges. Furthermore, Shao et al. (34) demonstrated that the distance of MVI from the tumor was associated with survival and recurrence in ICC patients, and it seemed that there was an incremental worsening DFS and OS as distance increased.

In this study, the radiomics approach and conventional clinical-radiologic method were used and compared to predict the presence of MVI in ICC patients. The two achieved comparable performance in the prediction of MVI. Furthermore, the nomogram combined radiomics signature and significant radiological factors achieved satisfactory discriminative ability, shown by an AUC of 0.886 in the training dataset and 0.80 in the test dataset. The radiomics features selected in this study were mainly wavelet transformation features consistent with other studies for MVI prediction (21, 22, 24). Among the six features in portal phase image signature, skewness measures the asymmetry of the gray level values distribution. Three GLCM features reflect the linear relation of gray level values or the local image homogeneity. GLSZM size zone nonuniformity reflects the image variability of size zone volumes. NGTDM strength is a measure of the primitives in an image. The wavelet transformation of raw images can further reflect more accurate spatial changes across multiple dimensions (26). Features included in this study varied from Zhou’s result (24), which may be attributed to the different image modalities analyzed.

From the comparison results of baseline characteristics, we found that the number of liver segments invaded, tumor size, tumor contour, age, PLT, satellite nodules, lymph node metastasis, arterial hypo-enhancement showed a significant difference between the MVI group and non-MVI group in the training dataset. However, only tumor contour, satellite nodules, and arterial hypo-enhancement were selected as significant risk factors by multiple regression. Tumor morphology is a key feature for MVI prediction both in HCC and ICC. It seems that MVI-negative patients are inclined to have a well-defined, spherical, oval tumor contour, whereas those with MVI tend to show irregular, blurry or infiltrative contours (18, 19, 35, 36). Our results are in agreement with these reports. In terms of satellite nodules, micro-metastases invade into the portal venous system and subsequently spread to the tumor-bearing portal territories and eventually develop into microsatellite nodules (37, 38). Such a mechanism has long been accepted as the main cause of intrahepatic metastasis and postoperative recurrence (39, 40). Satellite nodules are common in ICC patients, and the incidence is reported as high as 30% (41–43). The enhancement pattern of the mass-forming ICC in the hepatic arterial phase of CECT could be classified into three types: the hypo-enhancement, the rim-enhancement, and the hyper-enhancement (44). Several studies suggest that the different enhancement patterns of mass-forming ICC show varied prognosis, and the hypo-enhancement is often associated with worse survival (44–46). Yugawa et al. (47) reported that among the three enhancement patterns, the lowest tumor microvessel density was found in the hypo-enhancement ICC and was often accompanied by larger tumor size, more frequent microvascular invasion, and a higher rate of intrahepatic and lymph node metastasis.

Our study has some limitations. Due to the highly aggressive behavior of ICC, only a low number of patients admitted to our hospital have the chance for surgical resection, thus leading to limited sample size in our study. Secondly, retrospective nature of the study may introduce inevitable selection bias. Thirdly, this is a single-center study with internal validation was performed and in the absence of external validation. Therefore, further refinements with prospective multicenter studies are needed to check out our results.

## Conclusion

The findings in this study verified that both radiomics analysis and radiologic factors have the potential for MVI prediction in mass-forming ICC patients. The advantage of radiomics is that it can detect microscopic structures and quantitatively measure the microscopic changes in tissue caused by disease. The radiologic method was convenient and fast and also demonstrated good diagnostic efficacy. The combined nomogram, which integrated radiologic factors and radiomics signature, further improved the predictive performance for MVI diagnosis.

## Data Availability Statement

The original contributions presented in the study are included in the article/**Supplementary Material**. Further inquiries can be directed to the corresponding author.

## Ethics Statement

The studies involving human participants were reviewed and approved by The Ethics committee of the Second Affiliated Hospital of Zhejiang University School of Medicine. The patients/participants provided their written informed consent to participate in this study.

## Author Contributions

FX and SY were responsible for the conception of the work. SW reviewed pathological pictures. FX, XLia and XLiu obtained the data. FX and LY segmented the images. FX and XLia analyzed the data. FX wrote the manuscript. SY critically revised the manuscript. All authors are accountable for the contents of this work. All authors contributed to the article and approved the submitted version.

## Funding

This study was supported by grants from the National Natural Science Foundation of China (No.81572975) and Key research and development project of science and technology department of Zhejiang (No.2015C03053).

## Conflict of Interest

The authors declare that the research was conducted in the absence of any commercial or financial relationships that could be construed as a potential conflict of interest.

## Publisher’s Note

All claims expressed in this article are solely those of the authors and do not necessarily represent those of their affiliated organizations, or those of the publisher, the editors and the reviewers. Any product that may be evaluated in this article, or claim that may be made by its manufacturer, is not guaranteed or endorsed by the publisher.
