# Supplementary material for: Radiomics Analysis of Contrast-Enhanced CT for the Preoperative Prediction of Microvascular Invasion in Mass-Forming Intrahepatic Cholangiocarcinoma
Source: Front Oncol. 2021 Nov 19;11:774117. doi: 10.3389/fonc.2021.774117 (PMC8640186; doi:10.3389/fonc.2021.774117)
Supplement: Supplementary file 1 [file Image_1.pdf]

Supplementary Figure.1. Boxplot diagrams of the inter- and intra- class correlation coefficients (ICCs) for radiomic features on the CT dataset

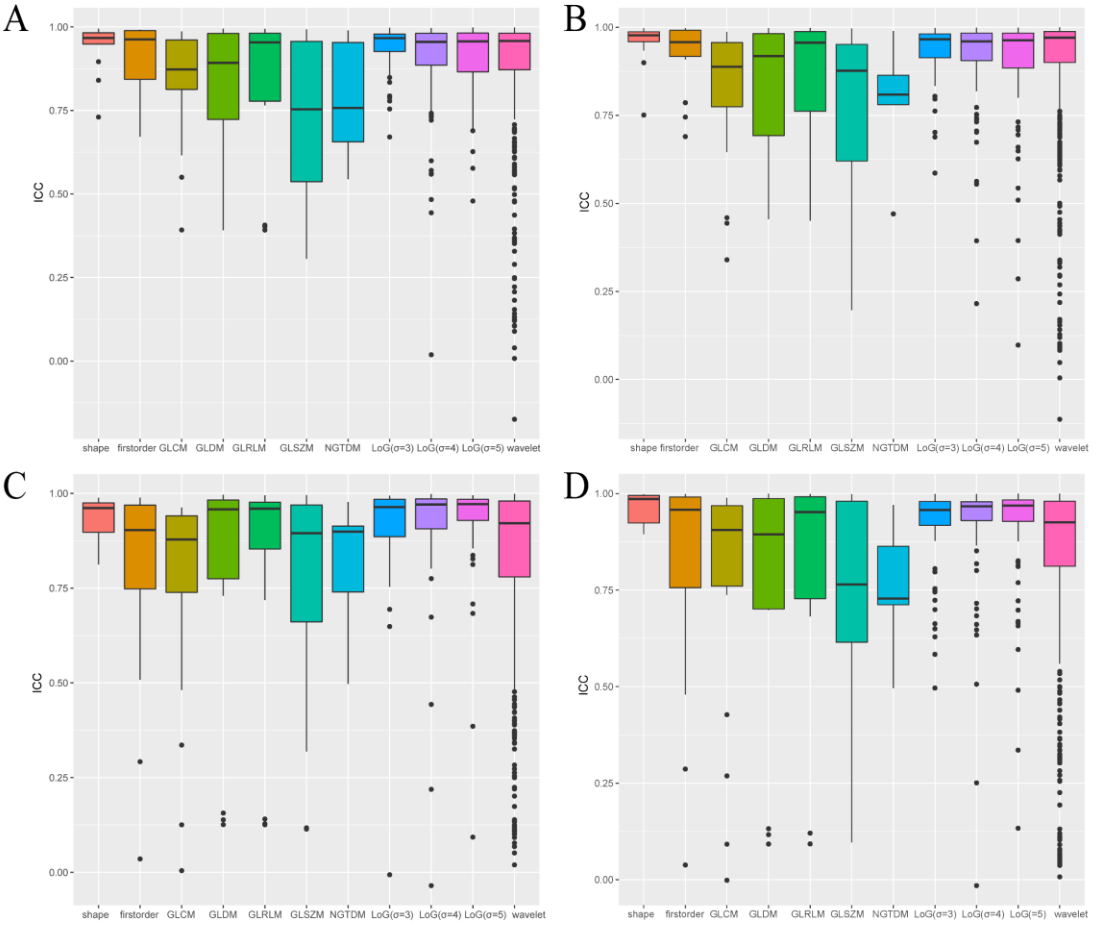

ICCs with eleven categories of features were calculated. The threshold level was established at 0.8. Features with ICC lower than 0.8 will be removed. For features exacted from portal phase image, the interobserver ICC (A) was ≥ 0.8, 0.5–0.79, < 0.5 for 83%, 13% and 4% of the features, respectively. The intraobserver ICC (B) was ≥ 0.8, 0.5–0.79, < 0.5 for 84%, 11% and 5% of the features, respectively. For features exacted from arterial phase image, the interobserver ICC (C) was ≥ 0.8, 0.5–0.79, < 0.5 for 77%, 15% and 8% of the features, respectively. The intraobserver ICC (D) was ≥ 0.8, 0.5–0.79, < 0.5 for 79%, 14% and 7% of the features, respectively. For features exacted from portal phase image, 226 features were excluded and 904 features were kept. For features exacted from arterial phase image, 319 features were excluded and 811 features were kept.
