# Supplementary material for: Radiomics Analysis of Contrast-Enhanced CT for the Preoperative Prediction of Microvascular Invasion in Mass-Forming Intrahepatic Cholangiocarcinoma
Source: Front Oncol. 2021 Nov 19;11:774117. doi: 10.3389/fonc.2021.774117 (PMC8640186; doi:10.3389/fonc.2021.774117)
Supplement: Supplementary file 2 [file Image_2.pdf]

Supplementary Figure.2. The process of feature selection and radiomics signature Construction

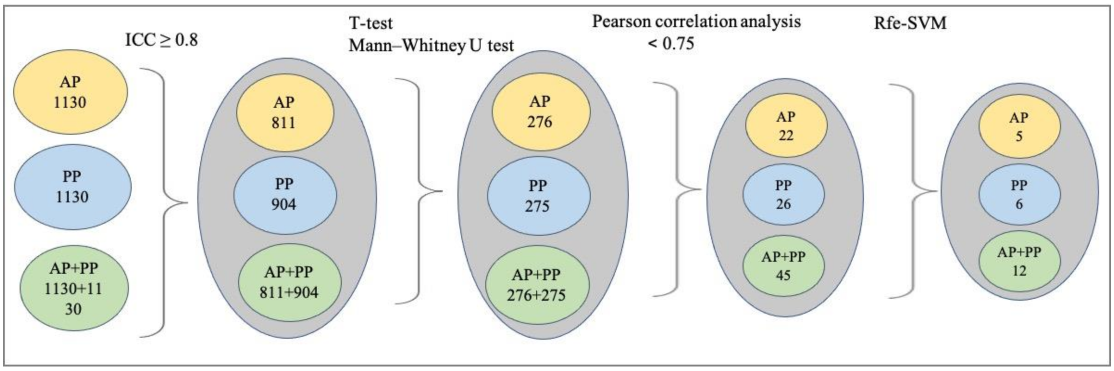

AP, arterial phase; PP, portal phase; ICC, intra- and inter-class correlation coefficients; Rfe-SVM, recursive feature elimination support vector machine.
