# Supplementary material for: Radiomics Analysis of Contrast-Enhanced CT for the Preoperative Prediction of Microvascular Invasion in Mass-Forming Intrahepatic Cholangiocarcinoma
Source: Front Oncol. 2021 Nov 19;11:774117. doi: 10.3389/fonc.2021.774117 (PMC8640186; doi:10.3389/fonc.2021.774117)
Supplement: Supplementary file 3 [file Image_3.pdf]

Supplementary Figure.3. The distributions of Rad-score in the training and test datasets.

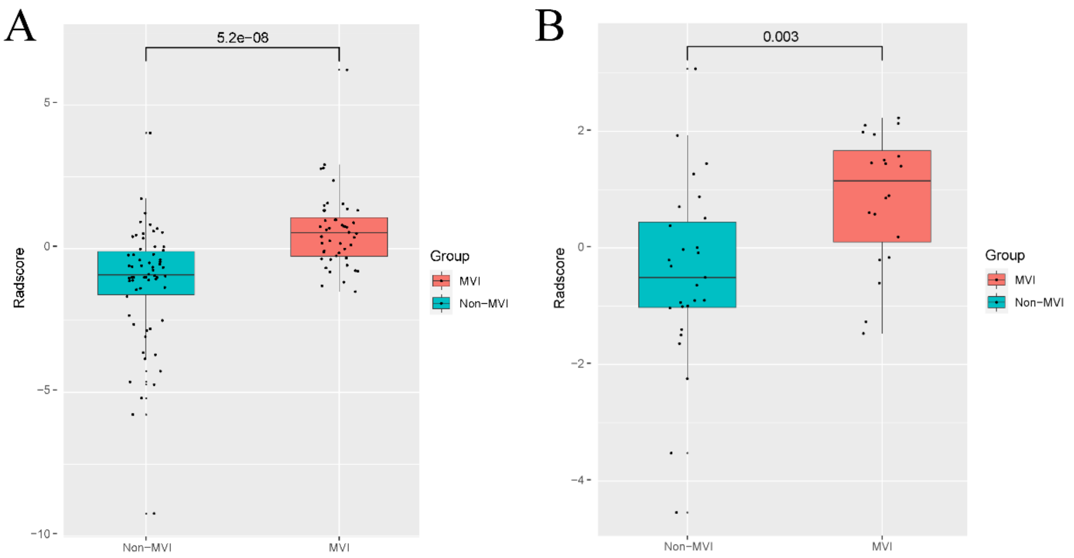

Boxplot diagrams show that the value of the Rad-score is significantly higher in patients with MVI in the training dataset (A) ($p < 0.001$) and the test dataset (B) ($p = 0.003$).
