# Supplementary material for: Radiomics Analysis of Contrast-Enhanced CT for the Preoperative Prediction of Microvascular Invasion in Mass-Forming Intrahepatic Cholangiocarcinoma
Source: Front Oncol. 2021 Nov 19;11:774117. doi: 10.3389/fonc.2021.774117 (PMC8640186; doi:10.3389/fonc.2021.774117)
Supplement: Supplementary file 4 [file Image_4.pdf]

Supplementary Figure.4. Decision curve analysis

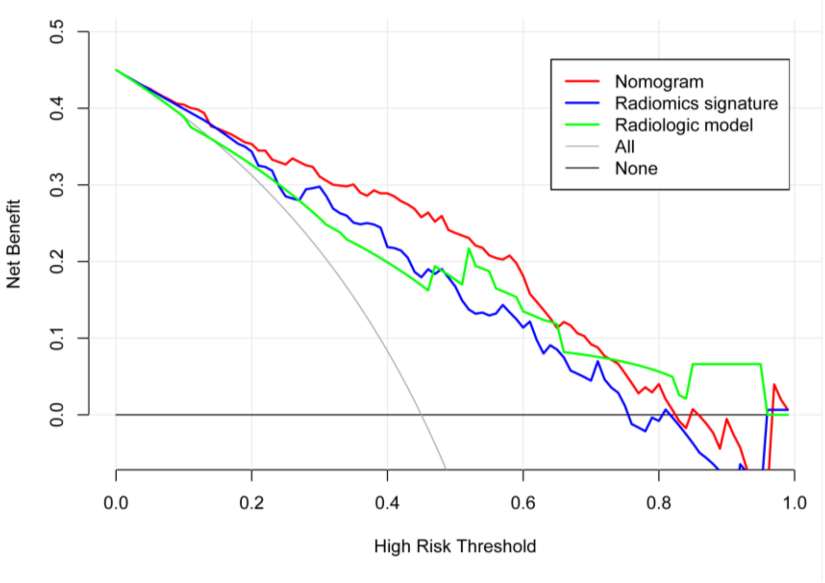

Decision curve analysis showed that the nomogram had a higher area under decision curves than Radiomics signature and radiologic model.
