# Supplementary material for: Radiomics Analysis of Contrast-Enhanced CT for the Preoperative Prediction of Microvascular Invasion in Mass-Forming Intrahepatic Cholangiocarcinoma
Source: Front Oncol. 2021 Nov 19;11:774117. doi: 10.3389/fonc.2021.774117 (PMC8640186; doi:10.3389/fonc.2021.774117)
Supplement: Supplementary file 5 [file Image_5.pdf]

Supplementary Figure.5. A representative case with MVI

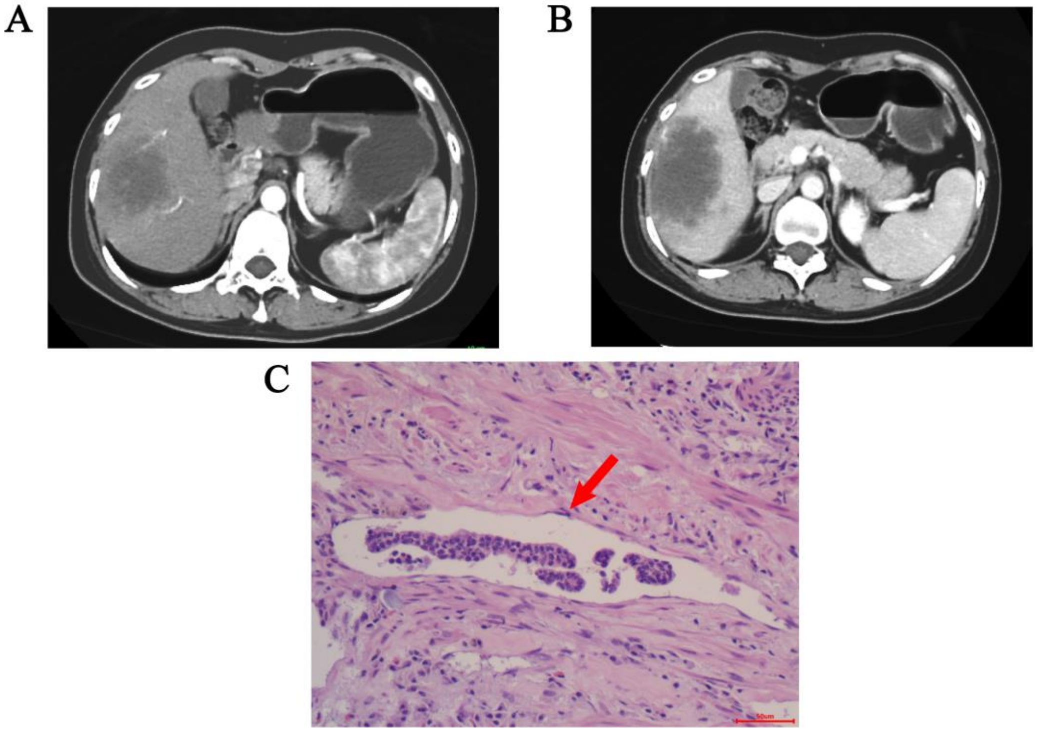

A 38-year-old woman was admitted due to upper abdominal discomfort and was diagnosed with mass-forming ICC. Laboratory tests showed: hepatitis B surface antigen positive, carcinoembryonic antigen (CEA), 1.2 ug/L; cancer antigen 19-9 (CA-199), 26.8 ug/L; AFP 4.2 ug/L. CECT showed that an approximately 4.9 cm × 8.5 cm low density shadow was found in the right interior lobe. In the arterial phase, the lesion shown a hypo-enhancement pattern, internal arteries, blurry margin. In the portal phase, the lesion remained to be low-density, satellite nodules could be observed around the lesion. The portal phase image Rad-score of the lesion was 0.063, and the nomogram model shown the probability for MVI was 90%. MVI (indicated with red arrow) was confirmed by microscopic examination.
