# Supplementary material for: Radiomics Analysis of Contrast-Enhanced CT for the Preoperative Prediction of Microvascular Invasion in Mass-Forming Intrahepatic Cholangiocarcinoma
Source: Front Oncol. 2021 Nov 19;11:774117. doi: 10.3389/fonc.2021.774117 (PMC8640186; doi:10.3389/fonc.2021.774117)
Supplement: Supplementary file 6 [file Presentation_1.pptx]

## Slide 1
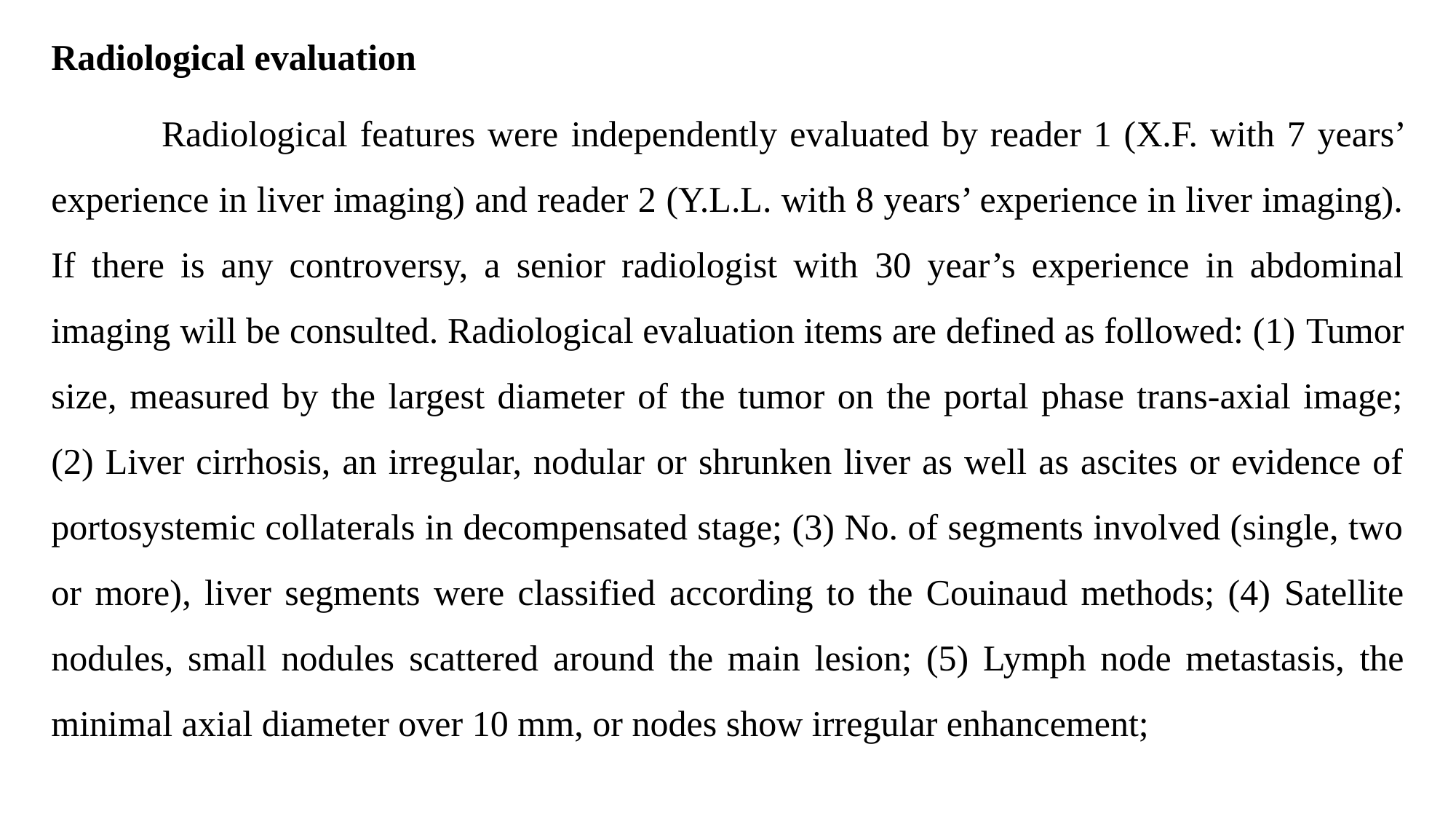

Radiological evaluation
 Radiological features were independently evaluated by reader 1 (X.F. with 7 years’ experience in liver imaging) and reader 2 (Y.L.L. with 8 years’ experience in liver imaging). If there is any controversy, a senior radiologist with 30 year’s experience in abdominal imaging will be consulted. Radiological evaluation items are defined as followed: (1) Tumor size, measured by the largest diameter of the tumor on the portal phase trans-axial image; (2) Liver cirrhosis, an irregular, nodular or shrunken liver as well as ascites or evidence of portosystemic collaterals in decompensated stage; (3) No. of segments involved (single, two or more), liver segments were classified according to the Couinaud methods; (4) Satellite nodules, small nodules scattered around the main lesion; (5) Lymph node metastasis, the minimal axial diameter over 10 mm, or nodes show irregular enhancement;

## Slide 2
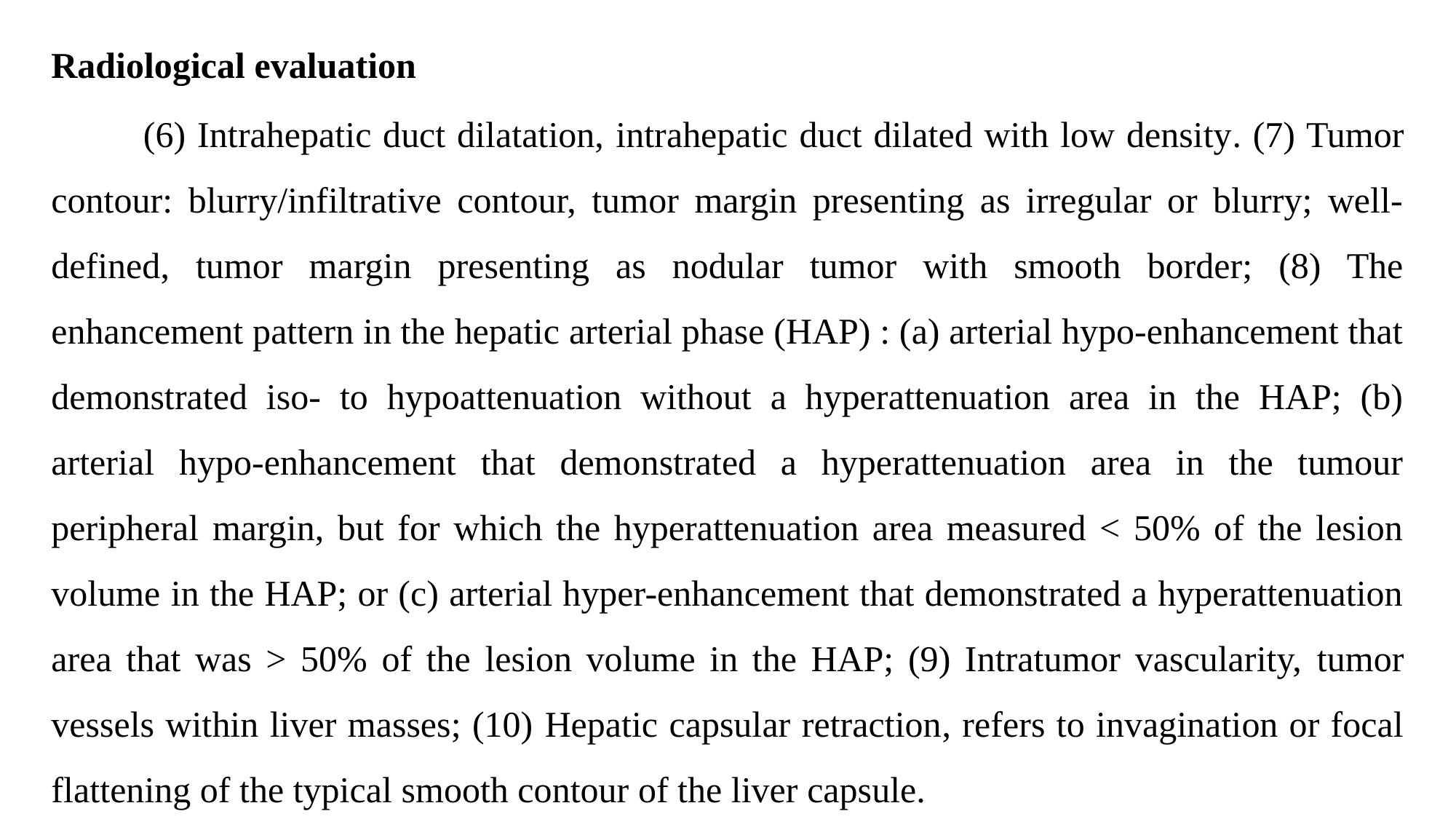

Radiological evaluation
 (6) Intrahepatic duct dilatation, intrahepatic duct dilated with low density. (7) Tumor contour: blurry/infiltrative contour, tumor margin presenting as irregular or blurry; well-defined, tumor margin presenting as nodular tumor with smooth border; (8) The enhancement pattern in the hepatic arterial phase (HAP) : (a) arterial hypo-enhancement that demonstrated iso- to hypoattenuation without a hyperattenuation area in the HAP; (b) arterial hypo-enhancement that demonstrated a hyperattenuation area in the tumour peripheral margin, but for which the hyperattenuation area measured < 50% of the lesion volume in the HAP; or (c) arterial hyper-enhancement that demonstrated a hyperattenuation area that was > 50% of the lesion volume in the HAP; (9) Intratumor vascularity, tumor vessels within liver masses; (10) Hepatic capsular retraction, refers to invagination or focal flattening of the typical smooth contour of the liver capsule.

## Slide 3
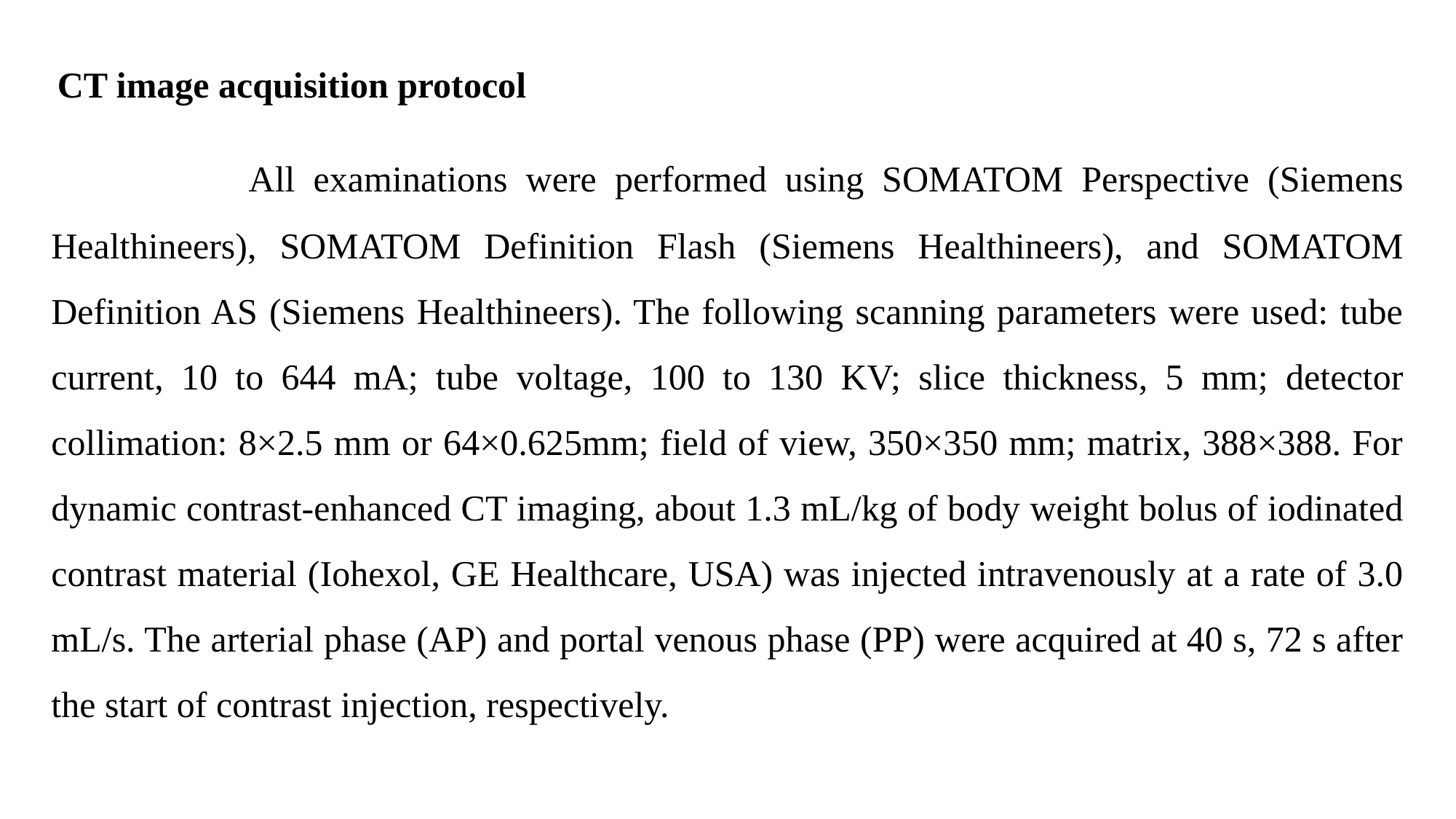

CT image acquisition protocol
 All examinations were performed using SOMATOM Perspective (Siemens Healthineers), SOMATOM Definition Flash (Siemens Healthineers), and SOMATOM Definition AS (Siemens Healthineers). The following scanning parameters were used: tube current, 10 to 644 mA; tube voltage, 100 to 130 KV; slice thickness, 5 mm; detector collimation: 8×2.5 mm or 64×0.625mm; field of view, 350×350 mm; matrix, 388×388. For dynamic contrast-enhanced CT imaging, about 1.3 mL/kg of body weight bolus of iodinated contrast material (Iohexol, GE Healthcare, USA) was injected intravenously at a rate of 3.0 mL/s. The arterial phase (AP) and portal venous phase (PP) were acquired at 40 s, 72 s after the start of contrast injection, respectively.

## Slide 4
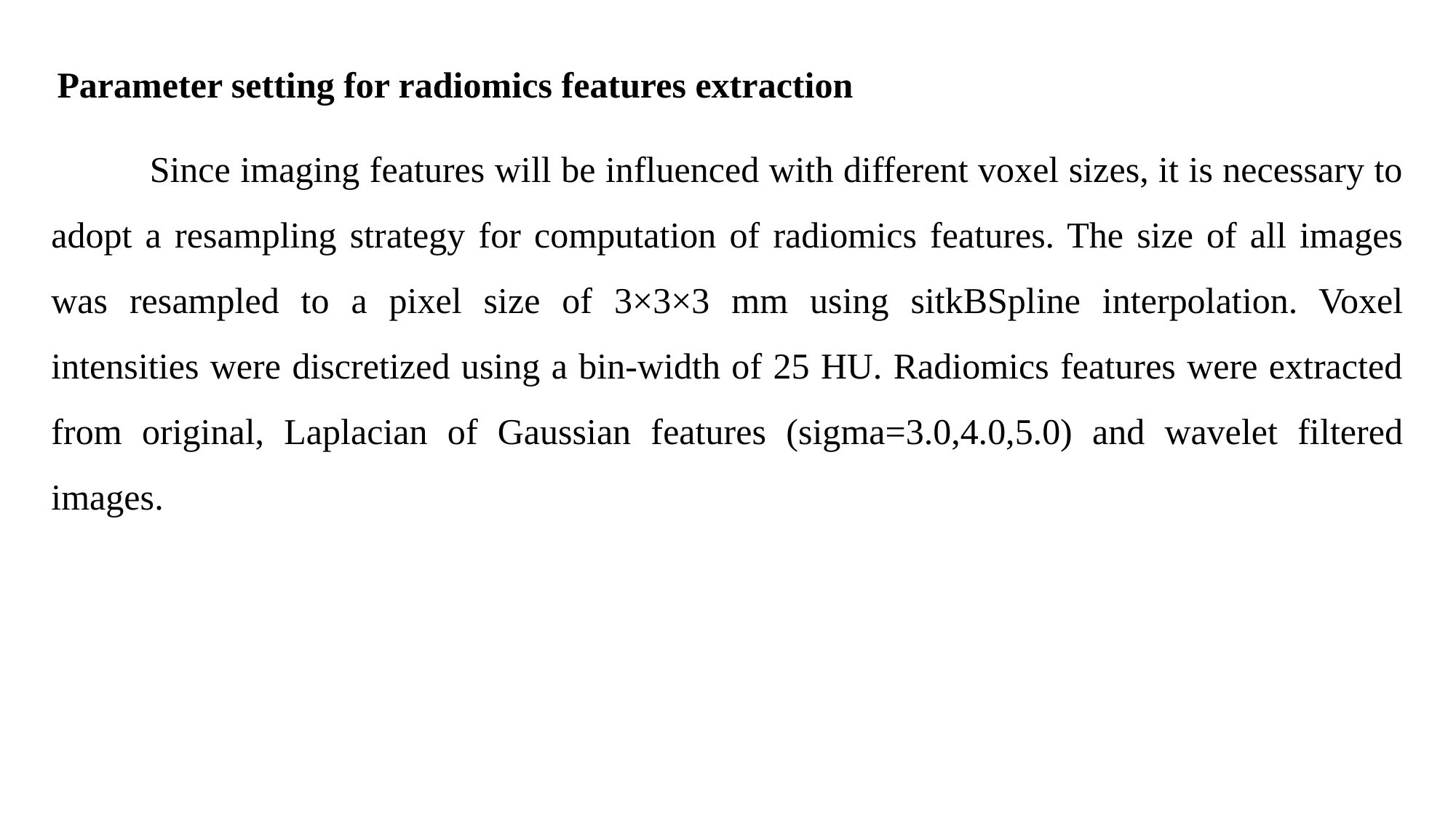

Parameter setting for radiomics features extraction
 Since imaging features will be influenced with different voxel sizes, it is necessary to adopt a resampling strategy for computation of radiomics features. The size of all images was resampled to a pixel size of 3×3×3 mm using sitkBSpline interpolation. Voxel intensities were discretized using a bin-width of 25 HU. Radiomics features were extracted from original, Laplacian of Gaussian features (sigma=3.0,4.0,5.0) and wavelet filtered images.

## Slide 5
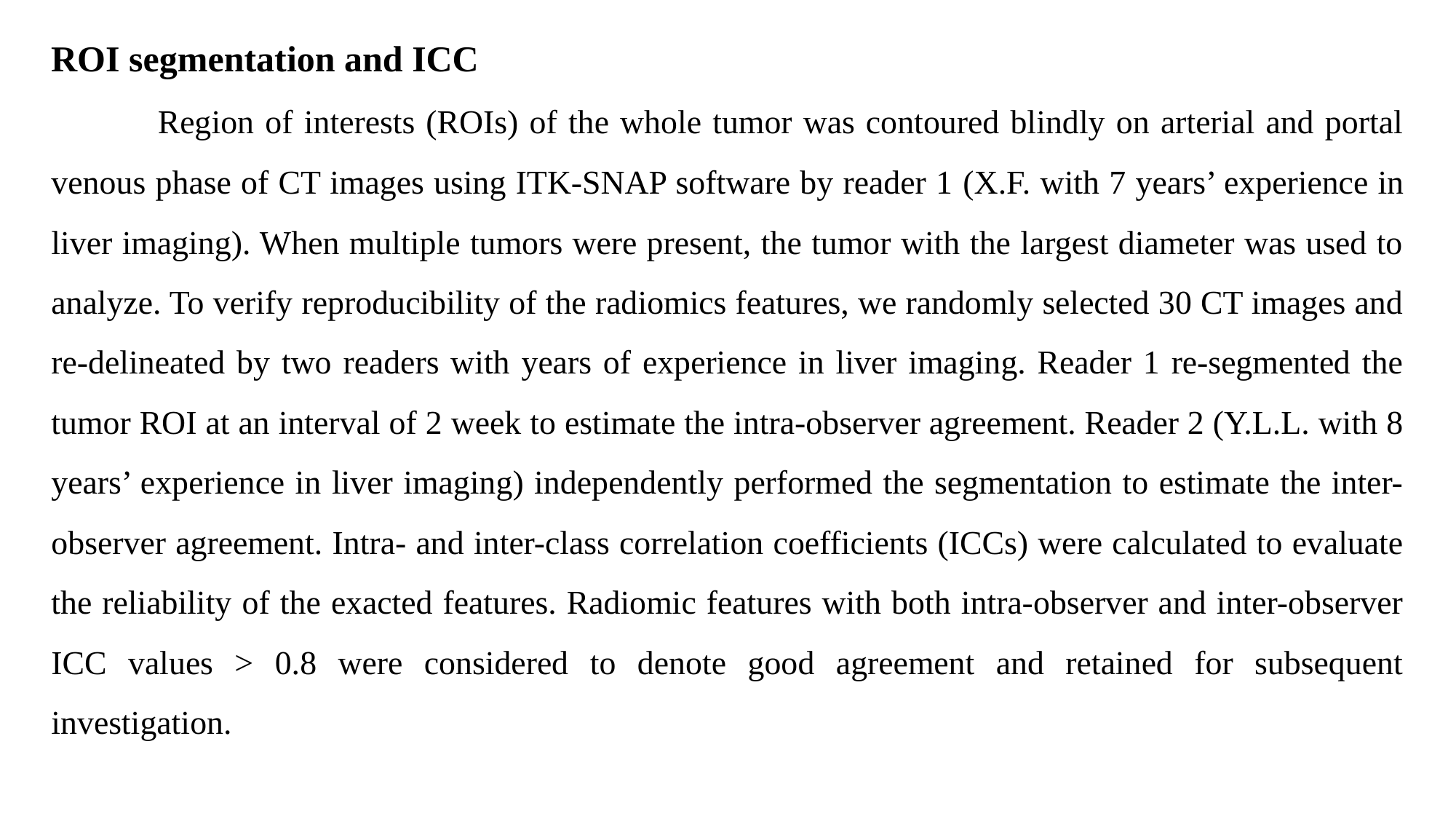

ROI segmentation and ICC
 Region of interests (ROIs) of the whole tumor was contoured blindly on arterial and portal venous phase of CT images using ITK-SNAP software by reader 1 (X.F. with 7 years’ experience in liver imaging). When multiple tumors were present, the tumor with the largest diameter was used to analyze. To verify reproducibility of the radiomics features, we randomly selected 30 CT images and re-delineated by two readers with years of experience in liver imaging. Reader 1 re-segmented the tumor ROI at an interval of 2 week to estimate the intra-observer agreement. Reader 2 (Y.L.L. with 8 years’ experience in liver imaging) independently performed the segmentation to estimate the inter-observer agreement. Intra- and inter-class correlation coefficients (ICCs) were calculated to evaluate the reliability of the exacted features. Radiomic features with both intra-observer and inter-observer ICC values > 0.8 were considered to denote good agreement and retained for subsequent investigation.

## Slide 6
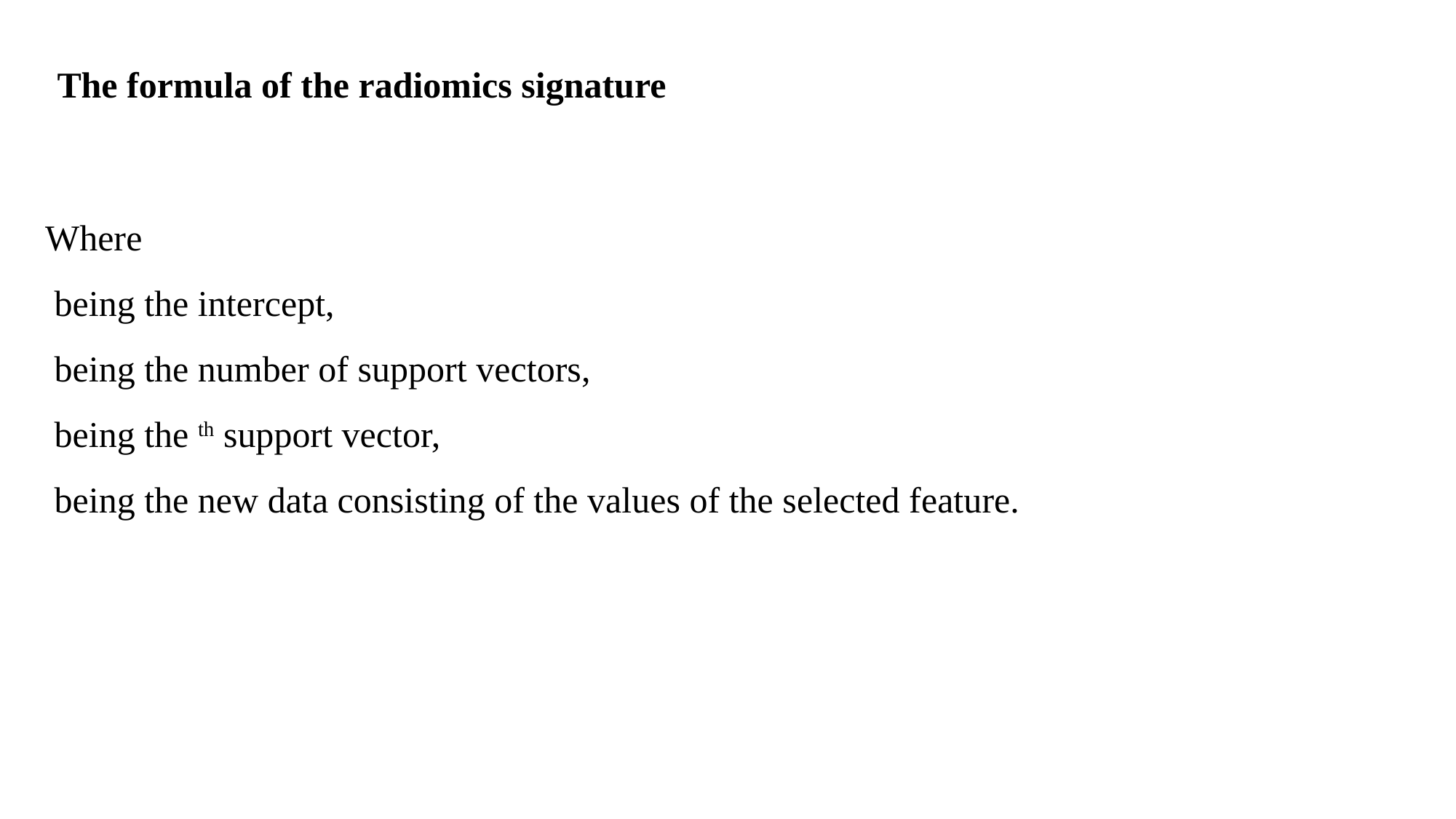

The formula of the radiomics signature
